# Developmental Changes in the Excitation–Contraction Mechanisms of the Ventricular Myocardium and Their Sympathetic Regulation in Small Experimental Animals

**DOI:** 10.3390/jcdd11090267

**Published:** 2024-08-29

**Authors:** Shogo Hamaguchi, Naoki Agata, Maika Seki, Iyuki Namekata, Hikaru Tanaka

**Affiliations:** Department of Pharmacology, Faculty of Pharmaceutical Sciences, Toho University, Funabashi 274-8510, Japan; shogo.hamaguchi@phar.toho-u.ac.jp (S.H.); naokiagat@gmail.com (N.A.); 3024002s@st.toho-u.ac.jp (M.S.); iyuki@phar.toho-u.ac.jp (I.N.)

**Keywords:** myocardium, development, action potential, Ca^2+^ handling, inotropy, adrenoceptors, sarcoplasmic reticulum, Na^+^-Ca^2+^ exchanger

## Abstract

The developmental changes in the excitation–contraction mechanisms of the ventricular myocardium of small animals (guinea pig, rat, mouse) and their sympathetic regulation will be summarized. The action potential duration monotonically decreases during pre- and postnatal development in the rat and mouse, while in the guinea pig it decreases during the fetal stage but turns into an increase just before birth. Such changes can be attributed to changes in the repolarizing potassium currents. The T-tubule and the sarcoplasmic reticulum are scarcely present in the fetal cardiomyocyte, but increase during postnatal development. This causes a developmental shift in the Ca^2+^ handling from a sarcolemma-dependent mechanism to a sarcoplasmic reticulum-dependent mechanism. The sensitivity for beta-adrenoceptor-mediated positive inotropy decreases during early postnatal development, which parallels the increase in sympathetic nerve innervation. The alpha-adrenoceptor-mediated inotropy in the mouse changes from positive in the neonate to negative in the adult. This can be explained by the change in the excitation–contraction mechanism mentioned above. The shortening of the action potential duration enhances trans-sarcolemmal Ca^2+^ extrusion by the Na^+^-Ca^2+^ exchanger. The sarcoplasmic reticulum-dependent mechanism of contraction in the adult allows Na^+^-Ca^2+^ exchanger activity to cause negative inotropy, a mechanism not observed in neonatal myocardium. Such developmental studies would provide clues towards a more comprehensive understanding of cardiac function.

## 1. Introduction

Small experimental animals are indispensable for the studies of the basic mechanisms of the heart and the creation of novel therapeutic agents. They are easy to handle, and their small body weight is of advantage for the in vivo screening of medicinal seeds. The hearts of small animals in general are known to have relatively high heart rates. Their myocardial contraction shares basic mechanisms such as the action potential and the Ca^2+^ transient, but difference exists in the process of repolarization and dependence on sarcoplasmic reticulum. The heart of the mouse is probably in the most extreme position among commonly used mammalian experimental animal species. The guinea pig heart has properties relatively close to those of larger animals and humans. The rat heart appears to have properties intermediate between the guinea pig and mouse. Such differences between animal species are small in the fetal stage and gradually appear during pre- and postnatal development. Understanding the similarities and difference among experimental animal species and developmental stages would provide insights into the factors involved in myocardial function. In this review, we will summarize the developmental changes in the myocardial excitation–contraction mechanisms and their sympathetic regulation, focusing on the ventricular myocardium of the guinea pig, rat, and mouse.

## 2. Action Potential Configuration and Membrane Currents in the Adult

**Figure 1:**
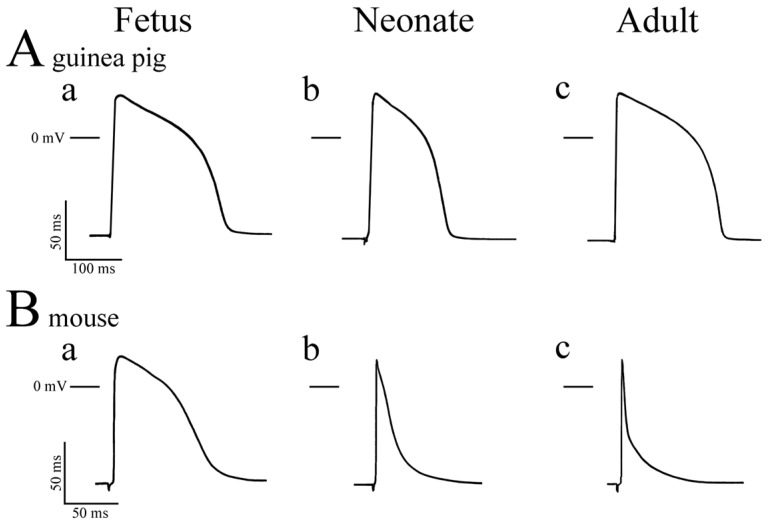
Action potentials of the guinea pig and mouse ventricle. Recordings were obtained in the guinea pig (**A**) and mouse (**B**) at fetal (**a**) neonatal (**b**) and adult (**c**) stages.

Animals of smaller size generally have higher heart rate and higher metabolic rate compared with larger animals and humans. The order among small experimental animals is mouse > rat > guinea pig [1,2,3,4,5,6,7,8]. While the maximum beating rate of the rat and mouse is as fast as 500 beats/minute and 800 beats/minute, respectively, the guinea pig heart has a beating rate of about 300 bpm, which is relatively close to larger animals and humans. The action potential of the guinea pig ventricular myocardium has an obvious plateau phase resembling those of larger animals (Figure 1(Ac)); the action potential duration is 200 to 300 ms. The plateau phase plays important roles in myocardial contraction, such as the trans-sarcolemmal influx of Ca^2+^ to trigger contraction and the formation of the refractory period to prevent arrhythmic excitation of the myocardium. Action potential duration is mainly determined by the balance between the L-type Ca^2+^ current (ICa) and repolarizing potassium currents. Voltage clamp analyses of ventricular cardiomyocytes revealed that the ICa is relatively similar among experimental animal species in terms of current density and kinetics [5]. On the other hand, there is a large species difference in repolarizing outward currents [9]. The major repolarizing current in the dog, rabbit, and guinea pig is the delayed rectifier potassium current (IK), which requires a few hundred milliseconds for maximum activation [10,11,12,13]; this delay causes a delay until the start of the repolarization and results in the typical action potential duration. Thus, the guinea pig has repolarization properties relatively similar to larger animals and humans, and is widely used in physiological and pharmacological studies oriented towards application in humans. 

The mouse and rat heart, which beats faster than the guinea pig, as mentioned above, shows rapid contraction and relaxation. Thus, the myocardium of these animal species should have characteristic excitation–contraction mechanisms to support its rapid contraction and relaxation. The mouse and rat ventricular myocardium have an action potential with a very short duration at depolarized membrane potentials and a late slowly repolarizing phase. The mouse ventricular action potential is very short, with an action potential duration at 50% repolarization of 3 to 5 ms (Figure 1(Bc)) [14,15,16,17,18,19,20,21,22]. Although very different from the human myocardium, the use of genetically modified mice enables exploration at the molecular level. Also, the dramatic change during development makes them suitable for studies such as the relation between action potential and inotropy (Table 1).

The repolarizing current in the mouse and rat is the transient outward current (Ito), which activates and inactivates much faster than IK. The Ito is composed of voltage-dependent K^+^ channel current and the Ca^2+^-activated Cl^−^ current [38]. The Ito in the ventricular myocytes of the mouse and rat has a high current density, which leads to rapid repolarization following the initial upstroke of the action potential. Following the rapid repolarization, a slow repolarizing phase, which is also referred to as the late plateau phase, is present in the mouse and rat ventricular myocardium. This phase is considered to reflect the inward current on the extrusion of Ca^2+^ by the sodium–calcium exchanger (NCX). The major role of NCX is the trans-sarcolemmal extrusion of Ca^2+^. NCX transports one Ca^2+^ ion in exchange for three Na^+^ ions, so the extrusion of intracellular Ca^2+^ results in the generation of an inward current that may cause membrane depolarization. In the case of the mouse and rat ventricle, this process occurs just after the rapid repolarization by Ito to form the late plateau. In fact, the late plateau phase is abolished by ryanodine, which inhibits Ca^2+^ release from the sarcoplasmic reticulum (SR) and is not observed in cells internally dialyzed with EGTA-containing solution [39]. Further, SEA0400, a highly selective inhibitor of myocardial NCX [40,41], as well as a low-Na^+^ extracellular solution, largely reduced the late plateau [18]. 

The action potential plateau at depolarized membrane potentials has functional roles in myocardial excitation, and the mouse and rat ventricular myocardium lacking this phase appear to have compensatory mechanisms. Firstly, a longer plateau results in a larger volume of depolarized myocardium at the excitation wavefront, which electrotonically supports forward propagation of the action potential. Shorter action potential duration of the mouse and rat action potential means less electrotonic support from behind at the excitation wavefront. We have observed that the maximum rate of rise of the mouse ventricular action potential is significantly larger than that of other species [17], which may indicate larger sodium current density in the mouse ventricular myocyte. A larger depolarizing current at the excitation wavefront would provide larger depolarizing power and might compensate for less electrotonic support from behind in this species. Secondly, Ca^2+^ entering the cell through the L-type Ca^2+^ channel during the plateau phase activates the contractile proteins and elicits myocardial contraction either directly or through triggering of SR Ca^2+^ release. The short action potential of the mouse and rat allows less Ca^2+^ to enter the cell. This appears to be compensated by elevated SR function, as described in the next section. Thirdly, action potential duration parallels the length of the refractory period, which prevents the myocardium from premature firing in response to arrhythmogenic stimuli from ectopic foci or re-entry circuits. The short action potential duration, and thus a short refractory period of the mouse ventricular myocardium, means that it is susceptible to arrhythmogenic stimuli. We notice that isolated mouse myocardial tissue preparations tend to show arrhythmic contractions when driven at low frequencies in vitro. However, this susceptibility to arrhythmogenic stimuli can probably be masked in the in vivo mouse heart by its high beating rate.

## 3. Developmental Changes in the Action Potential

The maximum rate of rise of the mouse ventricle gradually increases during pre- and postnatal development [19], which correlates with an increase in the expression of voltage-dependent Na^+^ channels [28,29]. While the adult ventricular myocardium of the mouse and rat has characteristic action potentials of short duration, the fetal and neonatal myocardium of these species have an action potential with a longer duration: developmental shortening is observed during pre- and postnatal development. We reported that the action potential duration of the mouse ventricular myocardium is longer than 70 milliseconds at fetal day 16, about 15 milliseconds in the neonate, and shortens to a few milliseconds in the adult [19] (Figure 1B). Similar results have been repeatedly reported in ventricular myocardium from pre- and postnatal mice [15,16,19,20]. The major cause of this shortening is the developmental increase in repolarizing potassium currents. The neonatal mouse ventricular myocytes have a much lower current density of the Ito than the adult. Instead, the IK is present, resulting in a longer action potential duration. A similar developmental shortening of the action potential reflecting an increase in Ito was observed in the pre- and postnatal rat ventricle [21]. Concerning ICa, the current amplitude [30,32], as well as its expression level [28,31] increases during development; the time course of the current appears to be unchanged. Developmental changes in the Ca^2+^-dependent inactivation of the ICa has not been reported, and experimental evidence for and against the presence of such mechanisms have been reported [42,43,44].

In the guinea pig, shortening of the ventricular action potential is observed during the late-fetal and early-postnatal period [10]. Action potential duration reaches its minimum at around 5 days after birth, and a gradual increase is observed until adulthood (Figure 1A). Voltage-clamp analysis of isolated ventricular myocytes revealed developmental increase in both IK and ICa, but the time course was different. Increase in IK occurred during the late-fetal period, while the increase in ICa occurred postnatally. This could explain the biphasic developmental change in action potential duration in the guinea pig ventricle [11]. The ATP-sensitive potassium channel current was shown to be present in the guinea-pig ventricular myocardium as early as on the 35th day of fetal development [45,46], as confirmed by both shortening of the action potential by the ATP-sensitive potassium channel opener, cromakalim, and by direct measurement of the channel current by voltage clamp experiments. However, experimental hypoxia induced only a slight decrease in contractile force, as well as the action potential duration in the fetus. This was because the fetal myocardium was much less dependent on the oxidative metabolism than the adult, but rather dependent on ATP provided by glycolysis. Electron microscopic images of fetal myocardium have demonstrated the presence of glycogen granules [47]. Confocal microscopy revealed that the mitochondria were less abundant and tended to localize in the cell center, relatively far from the sarcolemma. Thus, the function of ion channels involved in the formation of the action potential is not only one of influencing each other electrically but also being influenced by intracellular metabolic properties.

## 4. Excitation–Contraction Mechanisms in the Adult

Ventricular myocytes from the adult mammalian heart have a well-developed T-tubular system throughout the cell. During the action potential plateau, Ca^2+^ influx through the sarcolemma and T-tubules triggers Ca^2+^ release from ryanodine receptors located on the adjacent SR membrane. This results in a higher Ca^2+^ concentration at the Z-band region of the ventricular myocyte only for several milliseconds during the early phase of contraction, and at about 10 milliseconds after the onset of action potential, Ca^2+^ concentration is uniform throughout the whole cytoplasm [48]. In contrast, in atrial cardiomyocytes, which lacks the T-tubular system, trans-sarcolemmal Ca^2+^ influx triggers Ca^2+^ release only at the subsarcolemmal SR. This Ca^2+^ triggers Ca^2+^ release from the neighboring SR, and a wave of Ca^2+^-induced Ca^2+^ release propagates towards the cell interior as a Ca^2+^ wave [48]. 

The Ca^2+^ for the initial triggering of the Ca^2+^-induced Ca^2+^ release is provided by the ICa, and the amount correlates with the APD at about 0mV or at 20% repolarization (APD_20_). In a voltage-clamped rat ventricular cardiomyocyte, the total Ca^2+^ influx elicited by an action potential with a duration at 0mV of 44 ms and 6 ms was 33 and 16 pC, respectively [49]. Under an action potential of short duration, the peak amplitude of the Ca^2+^ influx is large, due to the larger inward driving force, but the Ca^2+^ channel rapidly deactivates, resulting in less total Ca^2+^ influx. In the mouse ventricular myocardium, the action potential duration at 20% depolarization decreases during development from about 45 ms in the fetus to less than 3ms in the adult, indicating a decrease in the Ca^2+^ to trigger contraction [19]. The adult mouse ventricular myocardium probably compensates for this by its elevated SR function. When the effects of nicardipine and ryanodine on isolated myocardial tissue from mouse, rat, and guinea pig were compared to evaluate the relative role of trans-sarcolemmal Ca^2+^ influx and SR Ca^2+^ release, the potency order for negative inotropy nicardipine, which reflects dependence on trans-sarcolemmal Ca^2+^ entry, was guinea pig > rat > mouse [19,50,51]. This correlated with the species difference in action potential duration. On the other hand, the potency order for the negative inotropy by ryanodine, which reflects dependence on SR Ca^2+^ release, was mouse > rat > guinea pig. 

Hearts with higher beating rate require faster contraction and relaxation for sufficient refilling before the subsequent heartbeat. The rate of ventricular relaxation measured under the same conditions in our laboratory was in the order of mouse > rat > guinea pig [19,50,51], which was the same as the order of higher heart rate and lower heart weight. This could be accomplished by increasing Ca^2+^ release and uptake by the SR. Comparative studies with various mammals revealed hearts of smaller weight have a higher resting heart rate and higher SR Ca^2+^. The mouse heart, which had the smallest weight, had the highest beating rate and SR Ca^2+^ ATPase activity [3]. The potency of the negative inotropy of cyclopiazonic acid, which impairs SR function through inhibition of Ca^2+^ uptake into the SR by the Ca^2+^ ATPase, was also in the order of mouse > rat > guinea pig.

## 5. Developmental Changes in Excitation–Contraction Mechanisms

**Figure 2:**
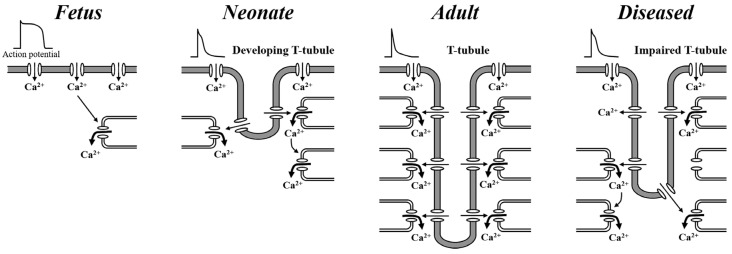
A schematic diagram of developmental and pathological changes in the excitation–contraction coupling of mouse ventricular cardiomyocytes. Fetus: The action potential triggers sarcolemmal Ca^2+^ influx through voltage-dependent Ca^2+^ channels, and a part of the Ca^2+^ diffuses towards the cell center and triggers Ca^2+^-induced Ca^2+^ release from Ca^2+^ release channels (ryanodine receptors) on the slightly present SR. Neonate: Partially developed T-tubular invaginations cause Ca^2+^-induced Ca^2+^ release from the coupled release channels on the SR. The released Ca^2+^ then triggers Ca^2+^ release from the neighboring Ca^2+^ release sites, uncoupled from the T-tubules. Adult: A well-developed T-tubular network enables simultaneous Ca^2+^ release from the SR in the entire cell on an action potential. Diseased: The T-tubule-SR coupling is partly disrupted and the SR Ca^2+^ release pattern resembles that in the immature myocardium.

The developing cardiomyocyte is not only smaller in size than the adult cardiomyocyte, but is also structurally very different. At the early stage of development, the t-tubules are very sparse and were present at the subsarcolemmal region mainly in a longitudinal orientation [52,53,54,55]. A progressive invagination of the t-tubules from the surface sarcolemma and alignment into a more transverse orientation takes place during postnatal development of the ventricular myocardium. The development of the t-tubular system is regulated by several key proteins including caveolin 3, junctophilin 2, ryanodine receptor, dysferin, bridging integrator-1 and protein phosphatase nonreceptor type 23 [53,56,57,58]. The SR is sparse in the fetus and neonate, and is present in the cell interior [19,50]; it expresses functional ryanodine receptors [59,60] localized to the Z-line prior to t-tubule formation [54,55]. Functional coupling of the t-tubules and the SR Ca^2+^ release units are formed progressively during postnatal development. In diseased myocardium such as that under heart failure, the integrity of the t-tubules as well as their coupling with the SR is impaired [54,61,62]. Increase in cardiac pre- and afterload has both increasing and decreasing effects on the t-tubular density, which appears to be related to compensatory mechanisms during heart failure [63]. The density of the t-tubular network in the adult tends to be denser in smaller animals than in larger animals [64,65]; among experimental animals, the mouse appears to have the highest t-tubular density. The t-tubular density of the mouse ventricular myocardium is very low in the fetal and neonatal myocardium and increases during the first and second postnatal week. The SR is present in the fetal cardiomyocyte and gradually increases during pre and early postnatal development [19]. These changes in cellular structure (Figure 2) and the concomitant changes in action potential configuration affects the functional properties of the murine myocardium. 

Concerning the developmental changes in the mouse ventricle, negative inotropic sensitivity to nicardipine and verapamil was significantly higher in the neonate than in the adult, which indicates that trans-sarcolemmal Ca^2+^ influx through the L-type Ca^2+^ channel becomes a progressively less-potent determinant of contractile force during postnatal development. The density of Ca^2+^ channels on the myocardial cell membrane as determined by specific binding sites for nicardipine was reported to increase two-fold during postnatal development [66]. The density of ICa on the cell membrane of the late-fetal mouse cardiomyocytes is comparable to that of the adult [67]. Thus, the developmental decrease in dependence of contraction on Ca^2+^ influx is not accompanied by a decrease in the number of functional Ca^2+^ channels themselves. It rather correlates with the shortening of the action potential duration due to developmental changes in the repolarizing potassium currents [15,16]. Similar shortening of the action potential duration [21] and a decrease in the inotropic sensitivity to nicardipine and verapamil have also been reported in the rat ventricular myocardium [50,68]. The postnatal changes appear to be larger in the mouse than in the rat. As the action potential duration of the neonatal mouse is much shorter than that of the neonatal rat, the corresponding developmental changes might be taking place earlier during the fetal period in the case of the mouse. In the case of the guinea pig, the Ca^2+^ channel blocker nicardipine was similarly effective in the ventricle from fetal, neonatal, and adult ventricle [51]. This appears to reflect the changes in action potential duration; the developmental shortening of the duration was less prominent in the guinea pig than in the mouse and rat (Figure 1). 

The negative inotropic sensitivity to ryanodine and cyclopiazonic acid was higher in the adult mouse than in the neonate, indicating that the role of SR in myocardial contraction and relaxation increases during postnatal development of the mouse [19]. Shortening of the late-plateau phase of action potentials by ryanodine, which reflects the amount of Ca^2+^ released from the SR, was observed in the adult mouse [19]. A three-fold developmental increase in the amount of the SR Ca^2+^ ATPase and its regulatory protein, phospholamban, has been reported in mouse myocardia [34]. The amount of both proteins was regulated at the transcriptional level, and the changes were accompanied by a developmental increase in the Ca^2+^ uptake velocity of SR vesicles. The SR Ca^2+^ ATPase determines the maximum uptake capacity, while phospholamban negatively regulates its sensitivity; the coordinated regulation of the expression levels of these two SR proteins may be necessary for maintaining Ca^2+^ homeostasis in the developing heart. Changes in cell morphology related to Ca^2+^ handling also take place during development. Fluorescence confocal microscopy revealed that the SR increases progressively during pre- and postnatal development of the mouse ventricle [19]. T-tubules were absent in the fetus and neonate, were present only in the subsarcolemmal region at 1 week after birth and were present throughout the cell by 2 weeks after birth. The amplitude of Ca^2+^ transients, as well as its ryanodine-sensitive component, increased gradually with age. In the neonate and 1-week-old mice, Ca^2+^ concentration at the cell center showed slower rise than in the subsarcolemmal region. In 2- and 4-week-old mice, Ca^2+^ concentration increased simultaneously across the entire width of the cell. These results indicated that the shortening of the action potential duration during the late-fetal period and the development of T-tubule-SR coupling during the second postnatal week largely contribute to the developmental increase in the dependence of contraction on SR function. Similar developmental increases in inotropic responses to ryanodine [50,68,69,70,71] and cyclopiazonic acid [72] have been observed in the postnatal rat. Developmental increases in negative inotropic sensitivity to ryanodine have also been reported in postnatal rabbit [73], late-fetal guinea pig [51], and middle-aged chick embryos, although the decrease in contractile force by ryanodine of these species was smaller than in the adult mouse ventricle. The effectiveness of ryanodine and cyclopiazonic acid on the SR in neonatal myocardia was confirmed with skinned myocardial fibers and rapid scanning confocal microscopy on cardiomyocytes.

## 6. Adrenoceptor-Mediated Inotropy in the Adult

**Figure 3:**
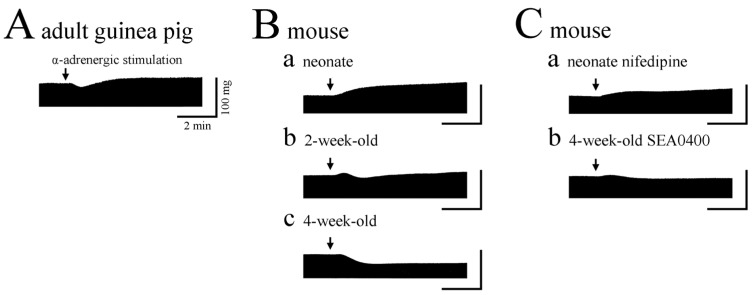
α-Adrenoceptor-mediated inotropy in the right ventricle. (**A**) Adult guinea pig showing positive inotropy. (**B**) Neonatal (**a**), 2-week-old (**b**), and 4-week-old mice (**c**). The inotropy changes from positive to negative during postnatal development. (**C**) Neonatal mice in the presence of nifedipine (**a**), 4-week-old mice in the presence of SEA0400 (**b**). The positive and negative inotropy was inhibited by nifedipine and SEA0400, respectively. Arrows indicate α-adrenergic stimulation by phenylephrine in the presence of propranolol.

The sympathetic nervous system is the major accelerator of the heart, and the positive chronotropic and inotropic responses to the transmitter, noradrenaline, are mainly mediated by β-adrenoceptor-induced increase in intracellular cAMP. The subsequent activation of protein kinase A results in several events, such as enhancement of Ca^2+^ influx through the sarcolemmal Ca^2+^ channel, acceleration of Ca^2+^ uptake into the SR, enhanced activity of the SR Ca^2+^ release channel, and changes in the Ca^2+^ sensitivity of the myofilaments. In addition to the β-adrenoceptor-mediated inotropy, α-adrenoceptors are present in the myocardium, and their stimulation generally produces no or weak positive inotropy through mechanisms different from those for β-adrenoceptor stimulation [25,74,75]). Sustained positive inotropy is observed in the rabbit, guinea pig, and rat ventricle, but in the guinea pig and rat, a transient negative phase is observed before the positive phase (Figure 3A). The adult mouse right ventricle showed a characteristic sustained negative inotropic response to α-adrenoceptor stimulation (Figure 3(Ba)) [28]. Similar sustained negative inotropy was observed in the mouse ventricle with endothelin-1 and angiotensin II [67]. The negative inotropic response to α-adrenoceptor stimulation in the mouse was accompanied by shortening of the slowly repolarizing phase of the action potential [17]. As this phase reflects extrusion by the NCX of Ca^2+^ released from the SR, it was probable that Ca^2+^ release from the SR was decreased by α-adrenoceptor stimulation. In fact, the amplitude of post-rest contraction, which reflects Ca^2+^ released from the SR, was attenuated after α-adrenoceptor stimulation. Attenuation of the Ca^2+^ transient by endothelin-1 was reported with mouse ventricular strips [76]. α-Adrenoceptor-mediated negative inotropy was not affected by ryanodine and cyclopiazonic acid. The time course of contraction and relaxation was not affected by α-adrenoceptor stimulation. These results indicated that α -adrenoceptor-mediated decrease in SR Ca^2+^ release was due to indirect effect through trans-sarcolemmal Ca^2+^ movements. α-Adrenoceptor-mediated negative inotropy was not affected by nicardipine, ouabain, and dimethylamiloride but was concentration-dependently inhibited by SEA0400, a selective inhibitor of NCX (Figure 3(Cb)) [17]. It was also attenuated under elevated external Ca^2+^ or decreased external Na^+^ conditions, which inhibit Ca^2+^ extrusion by the NCX. In voltage-clamped mouse ventricular myocytes, α-adrenoceptor stimulation increased the NCX current in both outward and inward directions. These results suggested that α-adrenoceptor stimulation decreased SR Ca^2+^ content through enhancement of Ca^2+^ extrusion by the NCX and resulted in negative inotropy. In isolated mouse ventricular myocytes excited at low frequencies, α-adrenoceptor stimulation was reported to reduce cell shortening, with no significant reduction in Ca^2+^ transient amplitude [77]. This suggests that factors other than the above-mentioned NCX-mediated decrease in Ca^2+^ transient amplitude, such as decrease in Ca^2+^ sensitivity of the contractile proteins [78], may also be involved in the α-adrenoceptor-mediated negative inotropy in the mouse ventricle.

## 7. Developmental Changes in β-Adrenoceptor-Mediated Regulation

Developmental changes occur not only in the excitation–contraction mechanisms, but also in the response to autonomic transmitters and various endogenous substances. The mouse heart starts to beat on the 9th day of fetal life [79], and its responsiveness to noradrenaline [80] and acetylcholine [81] is reported to increase rapidly during the late-fetal period. Further modifications of the response to transmitters occur during the early postnatal period. We have found that although noradrenaline produces a positive chronotropic and inotropic response during all stages of pre-and postnatal development, the sensitivity (*p*D_2_ values) to noradrenaline is higher in the neonatal mouse than in the adult [82]. This phenomenon was also observed in the developing rat, and appeared to be related to the increase in autonomic innervation, which occurs postnatally [83,84]. Increase in the sympathetic innervation of the ventricle was confirmed with the increase in inotropic responsiveness to tyramine, which releases noradrenaline from sympathetic nerve terminals. We investigated the mechanisms for the 10-fold postnatal decrease in the inotropic sensitivity to noradrenaline of the mouse and rat ventricle and found that two different mechanisms were involved. Firstly, increased uptake of the transmitter by sympathetic nerve terminals decreases the effective transmitter concentration at the receptor site and results in an apparent decrease in the sensitivity to applied noradrenaline (pre-junctional mechanism). This was confirmed by the observation that the sensitivity to noradrenaline is increased in the adult ventricle in the presence of desipramine, which inhibits neuronal uptake of the transmitter; this effect of desipramine was not observed in the neonate. Secondly, there was a decrease in the sensitivity to β-adrenoceptor-mediated intracellular mechanisms (post-junctional mechanisms). This was suggested by the observation that the sensitivity to noradrenaline was higher in the neonate than in the adult when compared in the presence of desipramine and that the sensitivity to isoproterenol, which is not taken up into nerve terminals, was also higher in the neonate. Such developmental decrease in sensitivity to noradrenaline was also observed in the late-fetal and neonatal rat heart, and both pre- and post-junctional mechanisms were involved. As no developmental change in sensitivity to dibutyryl cAMP and forskolin was observed, the developmental decrease in sensitivity in the rat heart to β-adrenoceptor stimulation was attributed to changes in the mechanisms prior to cAMP, such as β-adrenoceptor density and G-protein-mediated coupling of the receptor to adenylate cyclase [85]. The observed parallelism between the developmental increase in innervation and the decrease in post-junctional sensitivity to β-adrenoceptor stimulation suggested the presence of a causal relationship. Surgical [86] and chemical [87] denervation of the rat heart resulted in elevated post-junctional sensitivity to noradrenaline. Isolation of the rat atria from sympathetic influence by organ culture resulted in high sensitivity to noradrenaline, similar to that observed in the fetal period prior to the development of sympathetic innervation; this was reversed by application of the neurotransmitter, noradrenaline, in the culture medium, suggesting that constant stimulation of the β-adrenoceptor by endogenous transmitter is important for the maintenance of sensitivity [84]. These results indicate that sympathetic innervation exerts influence on the myocardium to regulate its sensitivity to the neurotransmitter. Such a relationship has also been suggested for the response of the rat vas deferens to α-adrenergic stimulation [88,89]. The developmental decrease in sensitivity to β-adrenoceptor stimulation was accompanied by both a decrease in receptor density and the coupling of the receptor with adenylate-cyclase [85]. This is similar to the case of heart failure, which is characterized with an impaired β-adrenoceptor-mediated inotropy accompanied by decreases in sarcolemmal β-adrenoceptor density (down-regulation) and coupling with intracellular mechanisms (desensitization) [90,91,92]. Such a condition of reduced β-adrenoceptor responsiveness is induced by the hyperactivity of the sympathetic nervous system to compensate for the reduced cardiac function. Experimentally, a similar condition of cardiac remodeling can be created by administration of β-adrenoceptor agonists [93]. 

## 8. Developmental Changes in α-Adrenoceptor-Mediated Regulation

As mentioned above, the adult mouse right ventricle shows a characteristic negative inotropic response to α-adrenoceptor stimulation. In contrast, the α-adrenoceptor mediated inotropy in mouse ventricle is positive during the first postnatal week [26]. It gradually decreases and is converted to negative by the third postnatal week. The α-adrenoceptor-mediated component contributes to the overall inotropic response to noradrenaline, both in the neonate and adult. Inotropic response to endothelin-1 and angiotensin II was also positive in the neonate, and developmentally converted to negative. As the developmental time course of the conversion of α-adrenoceptor-mediated inotropy from positive to negative paralleled the increase in sympathetic innervation density, we performed chemical denervation of the postnatal mouse to clarify whether there is any causal relationship [94]. The conversion of inotropy from positive to negative was not inhibited, but the sensitivity of the response to α-adrenoceptor stimulation was rather increased in denervated myocardia. These findings provide additional evidence for the above-mentioned view that sympathetic innervation is causally involved in the adjustment of the sensitivity of the effector organ to the neurotransmitter. 

Concerning the cellular mechanisms for the developmental conversion of α-adrenoceptor-mediated inotropy from positive to negative, we are considering the positive and negative components of inotropy separately, and both appear to be related to the developmental shortening of the action potential. As mentioned above, the negative inotropy in the adult could be attributed to enhanced NCX leading to the reduction of intracellular Ca^2+^ involved in contraction. NCX counter-transports three Na^+^ ions for a single Ca^2+^ ion, and the direction and amount are determined by the trans-sarcolemmal concentration gradient of these ions and the membrane potential. The short action potential in the adult mouse ventricle favors the NCX operating in the Ca^2+^ extruding direction, so that enhancement of NCX by α-adrenoceptor stimulation results in a reduction of intracellular Ca^2+^. Such a mechanism would be less favored in the neonatal myocardium, which has a longer action potential duration. On the other hand, the α-adrenoceptor-mediated positive inotropy observed in the neonate is mediated by enhanced influx of Ca^2+^ through Ca^2+^ channels [27]. The positive inotropy was not affected by SEA0400, or by low Na^+^ extracellular solution, but was significantly reduced by nifedipine (Figure 3(Ca)), which inhibits Ca^2+^ influx through Ca^2+^ channels. In voltage-clamped cardiomyocytes from the neonatal ventricle, α-adrenoceptor stimulation results in an increase in the Ca^2+^ channel current amplitude with no changes in the voltage for activation. The outward potassium current was not affected by α-adrenoceptor stimulation. The positive inotropy was accompanied by an increase in action potential duration, which appeared to be an enhancing factor for the inotropy. The observation that pharmacological inhibition of the action potential prolongation by cromakalim, an ATP-sensitive K^+^-channel opener, significantly reduced the positive inotropy, supports this view. Such a mechanism is less favored in the adult myocardium in which an extremely strong repolarization takes place. Thus, the developmental change in inotropy is closely related to the developmental change in excitation–contraction mechanisms.

## 9. Resemblance of the Diseased Myocardium to the Developing Myocardium

The phenotypes of diseased myocardium appear to resemble those of the developing myocardium [95,96]. In the failing myocardium, the structure of the t-tubules and the SR that formed gradually during development are partly disrupted. The effective coupling between the t-tubule and the SR decreases. This results in a less-synchronized release of Ca^2+^ from the SR; some of the Ca^2+^ release channels are activated not directly by the influx Ca^2+^ from the t-tubules, but by the Ca^2+^ released from the neighboring Ca^2+^ release units [97], which is similar to the pattern in the neonatal myocardium [19,70]. In the diabetic myocardium of the mouse, these changes in cell structure are relatively modest, but a reduction in SR Ca^2+^ content is caused by the decreased expression of the SR Ca^2+^ pump and increased Ca^2+^ leak from the Ca^2+^ release channel, due to changes in its phosphorylation [98,99,100]. This results in a reduced amplitude and prolonged duration of the Ca^2+^ transient, decreased contractile force and slower relaxation of the myocardium [98,99,101]. This is accompanied by a prolongation of the action potential duration, which may be a mechanism to compensate for the decrease in contractile force. Further, the degree of α-adrenoceptor-mediated negative inotropy is reduced [102]. It appears as if the excitation–contraction properties of the myocardium reverted to the neonatal stage, as described above. To normalize the impaired SR function, the search for compounds which activate the SR Ca^2+^ pump is now in progress [101,103,104,105,106,107]. 

α-Adrenoceptor-mediated positive inotropy observed in heart failure is probably a compensatory mechanism to maintain cardiac output under impaired β-adrenoceptor mediated inotropy [25,75]. Among the three subtypes, α1A, α1B and α1D, the α1A subtype is most abundantly expressed in the cardiomyocyte both in mice and humans. Further, the abundance of the α1A subtype is maintained under heart failure. Thus, the mouse appears to be a suitable model to study human heart failure. α1A receptor-induced positive inotropy is accompanied by increases in cytoplasmic pH, the phosphorylation of the myosin light chain, and the Ca^2+^ sensitivity of the myofilaments. Under experimental heart failure induced by administration of a β-adrenergic agonist to mice, additional administration of an α-agonist induced an establishment of an α-adrenoceptor-mediated positive inotropy [93]. Thus, α-adrenergic stimuli not only induced positive inotropy, but also caused an enhancement in the α-adrenergic sensitivity itself. This was opposite in direction to the sympathetic nerve-induced decrease in sensitivity observed in the neonate mentioned above [94]. Thus, although positive inotropy is observed both in the neonatal and failing heart, the influence of α-adrenoceptor stimulation on the sensitivity of α-adrenoceptor-mediated inotropy appears to differ between the neonatal heart and the failing adult heart. Studies on the effectiveness of α-adrenoceptor agonists against heart failure are performed in mice and in human patients, and positive results are reported [108,109,110].

## 10. Functional Significance of the Developmental Changes

In this review, we summarized the action potential and excitation–contraction properties of the ventricular myocardium and their regulation through α- and β-adrenoceptor stimulation in small experimental animals, the guinea pig, rat, and mouse. The myocardium of the adult guinea pig had properties relatively close to those of larger animals and humans, such as a relatively low beating rate, an action potential with a plateau phase, a balanced dependence on contraction on both trans-sarcolemmal Ca^2+^ influx and SR Ca^2+^ release, and positive inotropic responses to both α- and β-adrenoceptor stimulation. In contrast, the myocardium of the adult mouse, and to a lesser extent, the adult rat, has different characteristics, such as high beating rate, extremely short action potential, and high dependence on SR Ca^2+^ release. The adult mouse myocardium showed a unique negative inotropic response to α-adrenoceptor stimulation. Such species difference was not so evident in the fetal stage. A shortening of the action potential and increase in the dependence on SR function took place during pre- and postnatal development. Such changes in the basic excitation–contraction properties of the myocardium appeared to be closely related to the changes in inotropic responses, especially to α-adrenoceptor stimulation in the mouse. The developmental changes, as well as the species difference, probably have physiological significance. For example, the high sensitivity to β-adrenoceptor stimulation of fetal myocardia would be of advantage in responding to circulating catecholamines in the absence of direct sympathetic innervation. The developmental conversion of α-adrenoceptor-induced inotropy from positive to negative in the mouse may be a mechanism to attenuate and optimize cardiac performance under the high beating rate in the adult. Studying the developmental changes in myocardial excitation–contraction mechanisms and their regulation would provide clues for a more comprehensive understanding of cardiac function. 

## Figures and Tables

**Table 1:** Developmental changes in the excitation–contraction related parameters and the expression of excitation–contraction-related proteins of mouse ventricular cardiomyocytes.

	Fetus	Neonate	Adult	References
T-tubule	none	immature	mature	[19,23,24]
Action potential duration	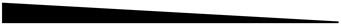			[15,16,19,20]
Ca^2+^ transient amplitude	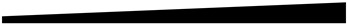			[19]
Nifedipine sensitivity in Ca^2+^ transient	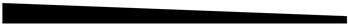			[19]
Ryanodine sensitivity in Ca^2+^ transient	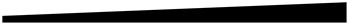			[19]
α-Adrenergic inotropism	positive	positive	negative	[18,25,26,27]
I_Na_	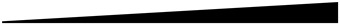			[28,29]
I_to_	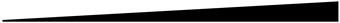			[16,20,28]
I_K_	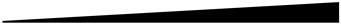			[15,16,20,28]
I_Ca-L_	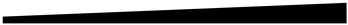			[28,30,31,32]
I_Ca-T_	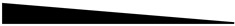			[28,33]
RyR2	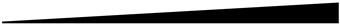			[28,30]
SERCA2	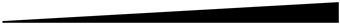			[28,30,34,35,36]
Phospholamban	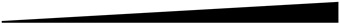			[28,30,34,35]
NCX	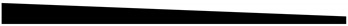			[28,30,34,37]

## Data Availability

No new data were created or analyzed in this study.
